# Conscious simultaneity with continuous motion: a measure-theoretic resolution of the hard problem

**DOI:** 10.3389/fnhum.2026.1809939

**Published:** 2026-05-20

**Authors:** John Sanfey

**Affiliations:** Independent Researcher, London, United Kingdom

**Keywords:** artificial consciousness, free-energy principle, general resonance theory, measure-theoretic limit, phase-amplitude coupling, quantum-classical incompatibility, temporal uncertainty principle, the hard problem

## Abstract

This paper addresses the problem of integrating phenomenal consciousness with physical laws by seeking to identify and define its function. The central claim is that the hard problem is caused by the same epistemic paradox that makes quantum and classical physics mutually incompatible: the *measure-theoretic limit*. It is logically impossible to explain the mechanism by which state transitions occur within continuous time except by using approximations, because the mathematics requires point-equivalent instants of zero duration, which cannot exist ontologically when time is continuous. Classical and quantum physics use mutually incompatible frameworks to model causality and overcome this *dt→0* limit. It is argued here that consciousness functions as an ontological workaround for this and all problems related to temporally extended information in continuous time, including sensory qualia. The *temporal uncertainty principle* (TUP) defines consciousness as a superposition of two contradictory temporal perspectives, synchronous and diachronic, within a single “now”. These perspectives interact recursively to reduce uncertainty to a point where further reduction is logically and physically impossible. This mechanism prevents computational paralysis when the system confronts unresolvable causal boundaries, and enables the generation of novel concepts and adaptive behaviours. The *bi-directional electromagnetic model* (BIDEM) postulates how the brain can achieve this mechanism within a *general resonance theory* (GRT) framework. By demonstrating how phase-amplitude coupling integrates two dimensionally orthogonal substrates, BIDEM enables diachronic information to act within simultaneously experienced instants. The model yields testable predictions for cross-frequency EM interactions and introduces a “simultaneity barrier” as the basis for an objective Turing test of artificial consciousness.

## 1 Introduction

Consciousness is not referenced in the fundamental laws of physics. Without a formal relationship to physical dynamics, it’s phenomenal aspect, P-consciousness (Block, 1995), cannot possess causal power, making it irrelevant to observable human behaviour (Chalmers, 1997; Chalmers, 1995). The aim of this paper is to establish an axiomatic basis for integrating consciousness and physics by identifying a first principle common to both. The central claim is that the subjective experience of simultaneity in continuous time biologically resolves the same problem of time that causes quantum-classical incompatibility. This establishes a fundamental postulate defining consciousness as a mandated physical function, and predicts a bi-directional electromagnetic theory of mind within the broader framework of general resonance theory (GRT) (Hunt and Schooler, 2019; Young et al., 2022).

A fundamental problem exists in mathematics known as the measure-theoretic limit (Halmos, 2013): the difficulty of assigning finite measures within something ontologically continuous. When physical reality is modelled as a sequence of discrete states within continuous time, causality becomes logically paradoxical (Russell, 2020). If a state is perfectly static, it must occupy an instant of zero duration (*dt → 0*) to preclude internal change. However, a zero-duration instant cannot possess the causal momentum necessary for transitioning to the next state. Conversely, if a state occupies a finite duration (*Δt > 0*), it inherently contains change when time is continuous, contradicting the premise of it being discrete. Therefore, causality and continuous time appear ontologically irreconcilable, and can only be managed by approximation techniques, or regularisations.

Classical and quantum physics use mutually contradictory abstractions to model state transitions in time. In general relativity, time is internal and dynamic, whereas in quantum field theory, it is external and fixed. The thesis of this paper is that consciousness functions as an ontological resolution to the paradox of integrating diachronic sensory and conceptual information within the zero-duration limit implicit in the experience of conscious ‘now-ness’. The phenomenon is defined here by the paradoxical co-existence of experiential simultaneity with diachronic flow, as a system capable of applying historical data during instants of experienced time without falling into the paralysis of algorithmic computation, as Penrose predicted (Penrose, 2016).

The phrase “something it is like” to be conscious (Nagel, 1980) captures the defining, temporal characteristic of P-consciousness. The phrase entails observer-observed simultaneity; there is no time gap between a state being experienced and the mind experiencing it. To explain this, this paper introduces the *temporal uncertainty principle* (TUP). The TUP defines consciousness functionally as a topological limit straddling two mutually contradictory explanatory paradigms. It represents a physical state of stationary uncertainty, beyond which further reduction of informational entropy is logically impossible.

While existing *general resonance theories* (Hunt and Schooler, 2019; Young et al., 2022; Schooler et al., 2011; Schooler and Riddle, 2024; Riddle and Schooler, 2024) describe consciousness as a nested hierarchy of synchronized electromagnetic (EM) fields, they struggle to explain the function of subjectivity without appealing to panpsychism, or succumbing to infinite regress without a specified nesting limit (Ramstead et al., 2023). The TUP, conversely, specifies a strict functional limit. To physically realize this limit, this paper proposes the Bi-Directional EM (BIDEM) theory. BIDEM describes two coupled systems with orthogonal dimensions and directions of informational flow, each driven to mirror the other by established physical and biological laws.

The paper positions consciousness as the formal bridge between abstract mathematical necessity and physical reality, and as a system with real causal agency. The temporal paradox of the measure-theoretic limit is described in section 2. Section 3 will review existing principles necessary for a comprehensive theory. The functional architecture of the TUP is covered in section 4, and the falsifiable biophysics of the BIDEM model are described in section 5.

## 2 Principle of observation equivalence and Turing test for consciousness

Descartes believed his ‘cogito’ argument established that awareness must exist *a priori* in order to think about it analytically (Descartes, 1996). Sartre replaced the term “a priori awareness” with “pre-reflective consciousness” (Sartre et al., 2022). Nowadays, P-consciousness is typically described in terms of “something it is like” to feel pain or see redness (Nagel, 1980). As mentioned above, this description entails *simultaneity* between something experienced and the mind experiencing it (Sanfey, 2023; Sanfey, 2024). It does not refer to simultaneity between two observed events which varies with the relative motion of observers as established in relativity theory.

### 2.1 Simultaneity and a Turing test for AI consciousness

The following argument demonstrates that experiential simultaneity has observable consequences, which makes it logically possible to integrate P-consciousness with physics.

The argument is this:

1. There is ‘something it is like’ to experience perceptual content simultaneously with the flow of continuous time.
2. There is ‘something it is like’ to consciously initiate an action within that same continuous instant.
3. To distinguish internally simulated data from external ontological reality, a system requires a zero-latency boundary where generated predictions and exogenic data intersect.
4. P-consciousness, by experiencing existential simultaneity, operates precisely at this zero-latency topological boundary.
5. A discrete, computational system is strictly bound by the latency of temporally sequential processing steps, making zero-latency intersection physically impossible.

*Conclusion*: the phrase ‘something it is like’ entails phenomenal simultaneity. Because it is bound by processing latency (*Δt > 0*), a non-conscious algorithmic system can never ontologically straddle the boundary between self-generated data and external reality, and can only compute temporally discrete approximations. Therefore, P-consciousness represents a distinct, non-computational physical function, providing the zero-latency boundary required to distinguish self-generated (autogenic) predictions from externally-sourced (exogenic) reality.

This deductive argument establishes a structural ‘Turing test’ for AI consciousness. Rather than measuring behavioural mimicry, the true test of consciousness is one of structural latency. Since current generative architectures operate as discrete state-transitions separated by processing time, they cannot fundamentally distinguish self-generated from exogenic data even if equipped with advanced exteroception and recursive self-monitoring. Only a system that experiences continuous diachronic flow within a synchronous ‘now’ can straddle this ontological barrier. Furthermore, this zero-duration simultaneity makes consciousness necessarily unobservable by sequential physical systems as a matter of principle.

This deductive argument confirms Penrose’s claim that consciousness must be non-computational (Penrose, 2016). Certain solutions are logically deducible by experiencing observer-observed simultaneity that are non-deducible to computational systems solely dependent on temporally sequential steps, including the possibility that ‘mind-stuff’ might exist independently of matter, which Descartes believed. As Penrose argued in relation to Gödel’s theorems, consciousness avoids paralysis by paradox. It can tackle ontological paradoxes creatively with inventive hypotheses. Consciousness endows the system with the degrees of creative and epistemic freedom necessary to navigate continuous change. A mechanism for how the brain achieves this will be proposed in section 5.

### 2.2 The measure-theoretic limit and simultaneous observation equivalence

An implicit assumption in science is that conscious subjectivity can be safely ignored provided physical laws and theories are based on replicable observations. However, if conscious perception is an active process rather than a transparent window, then, all observed phenomena *could* contain some fundamental artefact mistakenly assumed to be a fact about observed reality rather than the process by which it was observed. Analysis of the measure-theoretic limit shows this to be the case.

**Figure 1:**
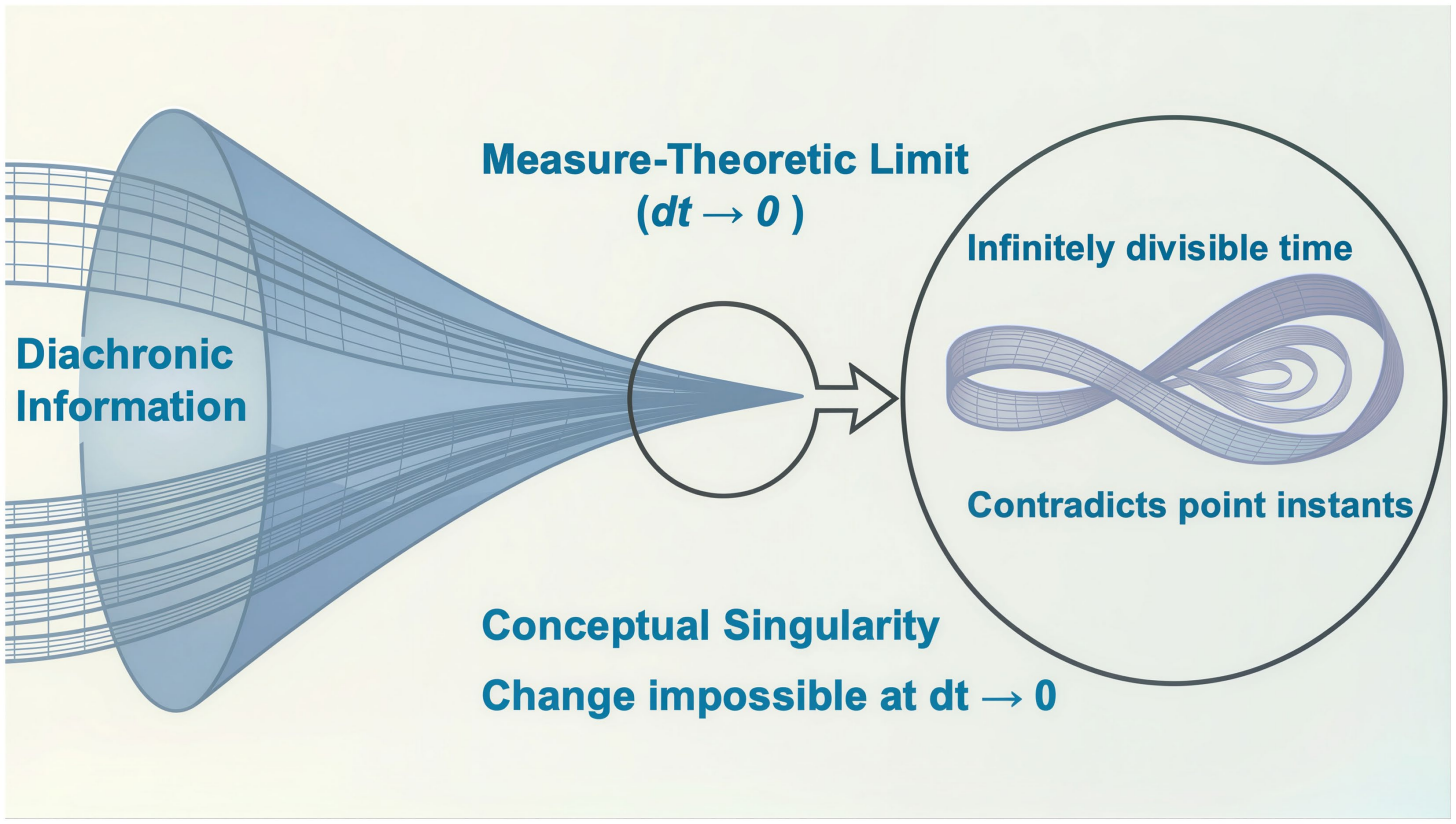
The diagram illustrates how physical states, mathematically forced to zero-duration instants (*dt→0*), cannot contain change, creating a fundamental explanatory paradox between causality and continuous physical motion. The central inset visualizes the resulting topological singularity: an infinite, unmeasurable regression within the point-equivalent instant, visually represented by an entangled, self-referential knot.

Standard models of neuro-information processing typically treat time as a continuous background variable, as do both branches of physics, albeit in different ways. This creates a fundamental, ontological paradox with causality. Any model of causality must compare before and after states, where each state has properties that are unchanging within a zero-duration instant. In both classical and quantum physics no two causally related states in an evolving system can share the same instant in time. But when time is continuous, the gap between states is infinitely divisible. As illustrated in Figure 1, the measure-theoretic limit forces the collapse of all diachronic data as *dt→0*. In a continuum, the journey to zero is infinite, or asymptotic, which is equivalent to Turing’s halting problem in computational terms. So, how do conscious observers manage the problem?

Every perceived instant of time is a tiny interval that contains continuous motion. So, given the assumption above, all observations contain both a cause and its effect. However, if time is continuous, and since cause and effect cannot occur simultaneously in physical dynamics, it follows that every conscious observation entails a retention of the immediate past. Furthermore, this retention requires action by the observer as Wolfram argues in his principle of computational irreducibility (Wolfram and Gad-el-Hak, 2003), (discussed further in 3.4). A simultaneous observing system cannot know *in advance* which earlier aspect of observed motion will form a meaningful temporally extended pattern at the end of its duration in time. A system that detects and explains invariance must apply rules that select those events most likely to be related to future effects as motion proceeds over time.

It follows that any mathematical inventions necessary for accurately explaining observations *can be considered* equivalent in function to this observer action (Sanfey, 2023; Sanfey, 2024; Sanfey, 2003; Sanfey, 2005). This is the *principle of simultaneous observation equivalence* (PSOE) within the framework of *abstract realism* (Sanfey, 2023; Kuhn, 2024). Techniques such as differential calculus and field theory tackle the same problem that awareness overcomes when making empirical observations. The measure-theoretic problem (Halmos, 2013), rooted in Russell’s analysis of the point-instant (Russell, 2020), is that causality cannot logically operate within point-equivalent instants because doing so requires the non-local integration of diachronic information. Both consciousness and physics *do* bridge former and latter instants of continuous time to create explanatory patterns within their *epistemic frames* of *reference*. However, epistemic systems in mathematical physics require abstract regularisation techniques, whereas conscious observation is an *ontological* solution that successfully avoids the computational halting problem at the measure-theoretic limit. How human brains achieve this ontological regularisation is examined in sections 4 and 5 below.

### 2.3 The foundation for a theory of P-consciousness

If these assumptions and arguments are correct, then certain postulates are potentially axiomatic. Firstly, the experience of existential simultaneity is a unique *physical* characteristic of P-consciousness; “physical” because it has observable consequences. Secondly, the mathematical description of any empirical phenomenon in physics must use abstract mechanisms whose function *can be considered* equivalent to the action of perceptual awareness in relating former and latter aspects of motion within an epistemic, explanatory framework.

It follows that P-consciousness can be produced by physical systems configured to perform this specific function. The function will be examined and defined further in sections 4 and 5. But first, a review of some important concepts and principles commonly used to relate consciousness and physics, and the various problems they encounter.

## 3 Time, information, observation and relational theory

This section will review those concepts and principles most relevant to the theory being developed here. For a more general review of consciousness theories see (Kuhn, 2024; Van Gulick, 2004).

### 3.1 The principles of stationary action and free-energy

The *principle of stationary action* (PSA) is a cornerstone of modern physics, spanning classical and quantum regimes. Action is defined as the time integral of the Lagrangian (kinetic minus potential energy), a functional representing the system’s dynamic state along a specific path in time. According to the PSA, the evolution of a physical system through its configuration space follows a path where action is stationary. This variational framework provides the mathematical bedrock from which fundamental equations of motion and conservation laws are derived. Although the principle is mathematically consistent across classical and quantum physics, its ontological implications differ. In classical physics it identifies a unique deterministic path, while in quantum theory, the stationary path is the sum of all possible histories.

In contrast to the PSA, the *free-energy principle* (FEP) is an informational principle. The concept of free-energy was introduced by Helmholtz in the nineteenth century, and relates the energy and entropy of a thermodynamically open system at constant temperature to the work available from the system (London, 1888). Using the relationship between entropy and information established by Shannon (Shannon, 1948), the FEP states that persisting systems must actively maintain models of both the environment and themselves in order to resist entropic mixing with their surroundings (Friston and Stephan, 2007; Friston, 2009; Friston, 2010; Friston et al., 2006; Parr et al., 2022). Variational free-energy can be defined as the upper bound on ‘surprise’ (or marginal likelihood) effectively functioning as the prediction error between raw sensory data and the system’s internal generative models.

The probabilistic foundation of FEP mandates the use of Bayesian inference, and is well suited to nested hierarchy models of systems-within-systems. Each sub-system is bounded by a *Markov blanket,* which exists mathematically as a set of variables that establishes conditional independence between internal and external states. Friston proved that the FEP is a corollary of the principle of stationary action (PSA), and that any self-organizing system that maintains its boundary (a Markov blanket) against entropy is mathematically minimizing its variational free energy, which is isomorphic to minimizing an action functional over time (Friston, 2013). Systems in the hierarchy receive probabilistic, top-down predictions for expected states, where predictions are defined as free-energy upper bounds, quantified using energy and entropy.

Being closely related to the second law of thermodynamics makes FEP scalable from molecular membrane receptors, ion channels, to organelles and cells, up to whole networks of neural connections within brains. However, there is a downside to having these thermodynamic foundations. The practicalities of calculating probabilistic states within nested hierarchies becomes completely intractable with increasing complexity, which forces FEP theory to use various heuristic approximations.

Var*iational free energy* is a family of mathematical techniques that approximate the sum of individual probabilities in complex interactions. For our purposes here, the most important technique conceptually, is derived from Solomonoff’s contribution to Kolmogorov complexity. He showed that the most probable explanation for complex data also has the shortest *algorithmic* description (Solomonoff, 1960).

### 3.2 Algorithmic information theory (AIT), Kolmogorov complexity and symmetry

In *algorithmic information theory* (AIT) (Chaitin, 1977), the informational content of a data string is equivalent to the length of the most compressed and self-contained representation of that string within the constraint of Turing’s halting problem. Following Solomonoff, AIT naturally incorporates FEP including variational free-energy because the shortest program capable of compressing entropic states without losing predictive power will produce states equivalent to Shannon information (Ruffini, 2017; Ruffini, 2007; Ruffini and Lopez-Sola, 2022). AIT thus precludes any need for formal calculation of Bayesian probability. As with FEP, modelling systems in AIT are separated from their immediate environment by Markov blankets, but here the coupling connection is *mutual algorithmic information* rather than ‘surprise’ (Ruffini and Lopez-Sola, 2022). AIT retains the power of FEP but is more practical for complex, hierarchical systems. Its compressive property will prove critically important in establishing the function of P-consciousness, and the mechanism by which it is achieved.

In addition to incorporating FEP’s relationship with the second law of thermodynamics, AIT claims a conceptual relationship to fundamental laws more generally, since physical laws can be characterised computationally as algorithms or rules governing the recursive transformation of initial states over time (Ruffini, 2017). This idea owes much to Wolfram’s emphasis on time in complex systems, discussed again in 3.4. Ruffini pointed out that the concept of transformation by the recursive application of rules is naturally related to symmetry laws, on which most physical laws depend (Ruffini and Lopez-Sola, 2022). *Symmetry* exists when a system remains invariant through some transformation, and it is always accompanied by a conserved quantity following Noether’s theorem (Noether, 1971).

Since its introduction in 2003 (Belkin and Niyogi, 2003; Cayton, 2008), the *manifold hypothesis* has become increasingly important in machine learning and neuroscience. The original idea, usually attributed to Riemann, is that the predictive core of a data set can often be reduced to a lower dimensional curved manifold within the data (Gonzalez-Castillo et al., 2023). The concept has proven useful for managing fMRI and EEG studies of the brain (Ruffini and Lopez-Sola, 2022; Gallego et al., 2018; Deco et al., 2025; Jirsa and Sheheitli, 2022; Pang et al., 2016; Ruffini et al., 2025; Albantakis, 2023; Runfola et al., 2025). Ruffini argues that manifold reduction can be considered equivalent to a conserved structure in the underlying dynamics and a generalisation of Noether’s theorem (Ruffini et al., 2025), which neatly integrates the Bayesian principles of FEP to potentially observable brain dynamics by making use of Solomonoff’s induction principle.

### 3.3 Observers and the causal structure of information

To what extent does the concept of ‘information’ imply an observer, conscious or otherwise? Tesler’s law, the first law of complexity, states that the complexity of a system cannot be eliminated, only re-allocated from a *user*-interface to the underlying mechanics (Saffer, 2007). This implies that removing the observer merely hides complexity within the mathematical machinery of the theory. However, the physicist Leonard Susskind suggests that information is more fundamental than anything else, that it exists independently of observers, and is even conserved in the singularity of a black hole (Susskind and Friedman, 2014). To J. A. Wheeler, on the other hand, reality only seems informational because nature is answering questions that observers ask by looking at it, making the universe ‘participatory’ (Wheeler, 2018). His *mutability principle* goes further by stating that no conserved property exists that cannot be unconserved if we look hard enough (Wheeler, 1973).

Several modern theories of consciousness are based on information, including *global workspace theory* (GWT) (Baars, 1997; Baars, 1993; Dehaene and Naccache, 2001). However, *integrated information theory* (IIT) warrants special attention for two reasons. Firstly, it claims the potential of resolving the hard problem eventually (Tononi and Koch, 2015), and secondly, it is a causal theory of P-consciousness.

IIT assumes that P-consciousness has causal power because its existence is more certain than whether matter exists independently of mind (Albantakis et al., 2023; Tononi, 2015). The theory postulates an identity between P-consciousness and an observable informational *structure* whose cause-and-effect properties are greater than the sum of its parts. It claims this explains both the privacy and causal power of consciousness (Tononi and Koch, 2015; Albantakis et al., 2023; Oizumi et al., 2014; Tononi, 2004; Tononi et al., 2016). Integrated information becomes a measurable physical quantity termed Φ (phi), defined as the maximally irreducible cause-and-effect power of the integrated system. Φ is calculated by assessing the impact of partitioning on the sum of a system’s parts. The theory has attracted its share of criticism (Sanfey, 2024; Albantakis, 2023; Barrett, 2014; Barrett, 2016; Barrett and Mediano, 2019; Doerig et al., 2019; Fleming et al., 2023; Frankish, 2023; Merker et al., 2022), but the most serious critique targets IIT’s identity between structure and causal power.

In IIT, the causal power of conscious systems is greater than the sum of its parts, and comes from information the system has about itself, encoded in *structures* that enable recursiveness. But structural rather than functional relationism means that the system would be conscious even when inactive, a claim readily acknowledged by IIT’s principal author (Tononi et al., 2022). Doerig’s *unfolding argument* (UA) highlights the consequences of any such structural identity by imagining the components of a recursive structure being replaced with feedforward elements that produce the same outcomes. Despite causing identical effects, the new system would no longer be conscious in IIT; a serious problem for a causal theory. Doerig’s argument is that causal structures can never explain consciousness (Doerig et al., 2019). A successful theory must define its function.

### 3.4 Time-asymmetry and causal history

Wolfram’s framework acknowledges the importance of time with his principle of *computational irreducibility*. “Observers like us,” also made of matter, cannot know *in advance* how any process not obviously simple will evolve over time (Wolfram and Gad-el-Hak, 2003; Wolfram, 2024). The only way to predict the evolution of a minimally complex, open system is by simulating the process computationally (Wolfram, 2024). Furthermore, human minds cannot compute individual movements of complex aggregations with the necessary speed. Instead, “observers like us” congregate equivalent parts into reduced representations with sufficient explanatory power (Wolfram, 2023). Both the second law of thermodynamics and causality’s arrow of time emerge from this coarse-graining (*equivalencing*), which Wolfram considers identical to the process of observation. Observers choose which causal history to select from the totality of all possible causal trajectories or “multiway graph.” One problem with this view is that conscious causal agency becomes illusory within a hidden, deterministic “block universe,” albeit one where the “hidden” part can be developed to accommodate quantum mechanics within a hypergraph model.

For Rovelli, on the other hand, time-asymmetry in thermodynamics exists because the past is fixed by macroscopically observable traces, whereas the future is open. Observers naturally align an arrow of time with observed motion from fixed states to those not yet known (Rovelli, 2022a,b). Although differing on the thermodynamic arrow of time, Rovelli agrees with Wolfram’s *causal arrow* because causality is thermodynamically rooted in two ways. Firstly, physical traces are empirically real, and secondly, they must also exist in the brains of observers (Rovelli, 2022a,b). In this view, consciousness is physical and its causal freedom describes the whole system’s independence from outside control. Consciousness is causally emergent *in practice* but not in principle, and results from complexity.

It should be noted that Rovelli’s macroscopic traces are only possible when interactions between objects dissipate heat. Dissipation is deeply related to the problem of integrating quantum and classical mechanics because interactions in isolated quantum systems are non-dissipative, an issue that assumes central importance in *quantum field theory* (QFT). In dissipative QFT, macroscopic traces are identical to the long-range correlations of spontaneous symmetry breaking in the underlying quantum dynamics of the system (Nambu, 1960). Anticipating the free-energy principle (FEP), Vitiello showed that when persisting dissipative systems create models of the environment to interact with, the mathematics of QFT can operate as if the system was thermodynamically closed (Vitiello, 2015; Vitiello, 1995; Vitiello, 2001). The interacting interface has two opposing time directions, doubling the degrees of freedom available to the whole system. In Vitiello’s *dissipative quantum model of brain* (DQMB), both information and the arrow of time are macroscopically ‘printed’ by spontaneous symmetry breaking in the underlying quantum dynamics at the mirroring interface, which endows consciousness with causal power. DQMB is considered further in section 5 below.

### 3.5 Emergence, non-locality and background independence

In IIT, consciousness is a causally emergent phenomenon (Oizumi et al., 2014; Marshall et al., 2018), and in AIT, a weakly emergent one (Ruffini et al., 2025). However, neither theory specifies the level of granularity where phenomenal causal power emerges from the ontologically continuous fields of fundamental physics (Barrett, 2014). The causal closure of physical laws demands that all *macroscopic* causal power must *supervene* on causality at the microscopic level (Kim, 2000; Kim, 1990), and without an axiomatic principle or law, neither IIT nor AIT can claim a foundational basis for conscious emergence.

Many of the early pioneers of quantum physics believed the new theory would explain consciousness. In particular, quantum non-locality evokes comparison with the *binding problem* of consciousness, in which distributed neural activity creates a multimodal, unified perceptual field (Von Der Malsburg, 1994). The empirical verification of Bell’s inequalities in the 1970’s triggered speculation that space, time and perhaps consciousness might be emergent properties of some deeper reality within a unifying cosmological theory. One of the biggest challenges for a unifying theory is that its fundamental fields should not exist on an external background. The theory should be *relational* or background independent, such that the universe does not occupy a physical container.

Wolfram (Wolfram and Gad-el-Hak, 2003; Wolfram, 2023; Wolfram, 2021), Rovelli (Rovelli, 1996, 2022a,b), Smolin (Cortês and Smolin, 2014; Smolin, 2013; Smolin, 2023; Smolin, 2020; Smolin and Verde, 2021) and others (Haramein et al., 2016), have developed fundamental relational theories that include consciousness, two of which allow for conscious causal power. Rovelli’s weak emergence has already been discussed, but Smolin’s *temporal naturalism* describes a panpsychist form of strongly emergent consciousness (Smolin, 2020). In this theory, all change, and hence time, is a relationship between fundamental objects defined in the theory as ‘views from ‘events.’ Events are objects consisting of conserved properties such as energy and momentum, which also contain ‘a view’ of their immediate causal history, where ‘immediate’ is purely temporal and spatially non-local (Smolin, 2023; Smolin, 2020). In this theory, the majority of events in nature are quantum states with ‘ensembles’ of causally equivalent ‘views’ that evolve in superposition according to the Schrödinger equation. However, conscious ‘events’ are those objects with only one causally possible view and exist in the macroscopic, classical domain. Conscious causal power is possible because the futures of these events are open, which in this theory also enables physical laws to evolve over time. As we shall see later, the biggest problem with Smolin’s panpsychist theory, and all theories that locate qualia at Planck scales, including the Hameroff-Penrose model, is how qualia can have any purpose in the timescale experienced as consciousness.

The essence of the PSOE, and its philosophy of abstract realism, is that every description in physics is formulated within abstract, *epistemic* backgrounds which are the structures of the theories themselves, and whose operation always requires some fundamental regularisation or approximation to resolve the measure-theoretic paradox. In a science based on observation, these axiomatic proxies can be considered functionally equivalent to how minds physically bridge past and future instants when perceiving *and contemplating* meaningful patterns in time. Wheeler and Susskind’s views of information are both correct, despite being contradictory. Information is both fundamental and abstract, because the process of observation cannot be eliminated from physics.

## 4 The basis for a physical law of consciousness

Metaphysics is the study of first principles on foundational issues. This section examines whether a unifying metaphysical principle exists that underpins both the hard problem and quantum-classical incompatibility. Since awareness is more certain than the independent existence of matter, such a principle should define the simplest possible ontological distinction in human awareness, and in a form that also defines the underlying cause of quantum-classical incompatibility.

The simultaneity between awareness and perceptual content makes it possible to define ‘physical’ phenomenologically as any experienced difference from nothing not knowingly caused by its experiencer, a definition that solipsists can agree with provided they recognise there is no ‘something it is like’ for them to *consciously* create reality (Sanfey, 2023). Existing physical laws remain unaffected by this definition of ‘physical’.

### 4.1 The self-referential identity of awareness

**Figure 2:**
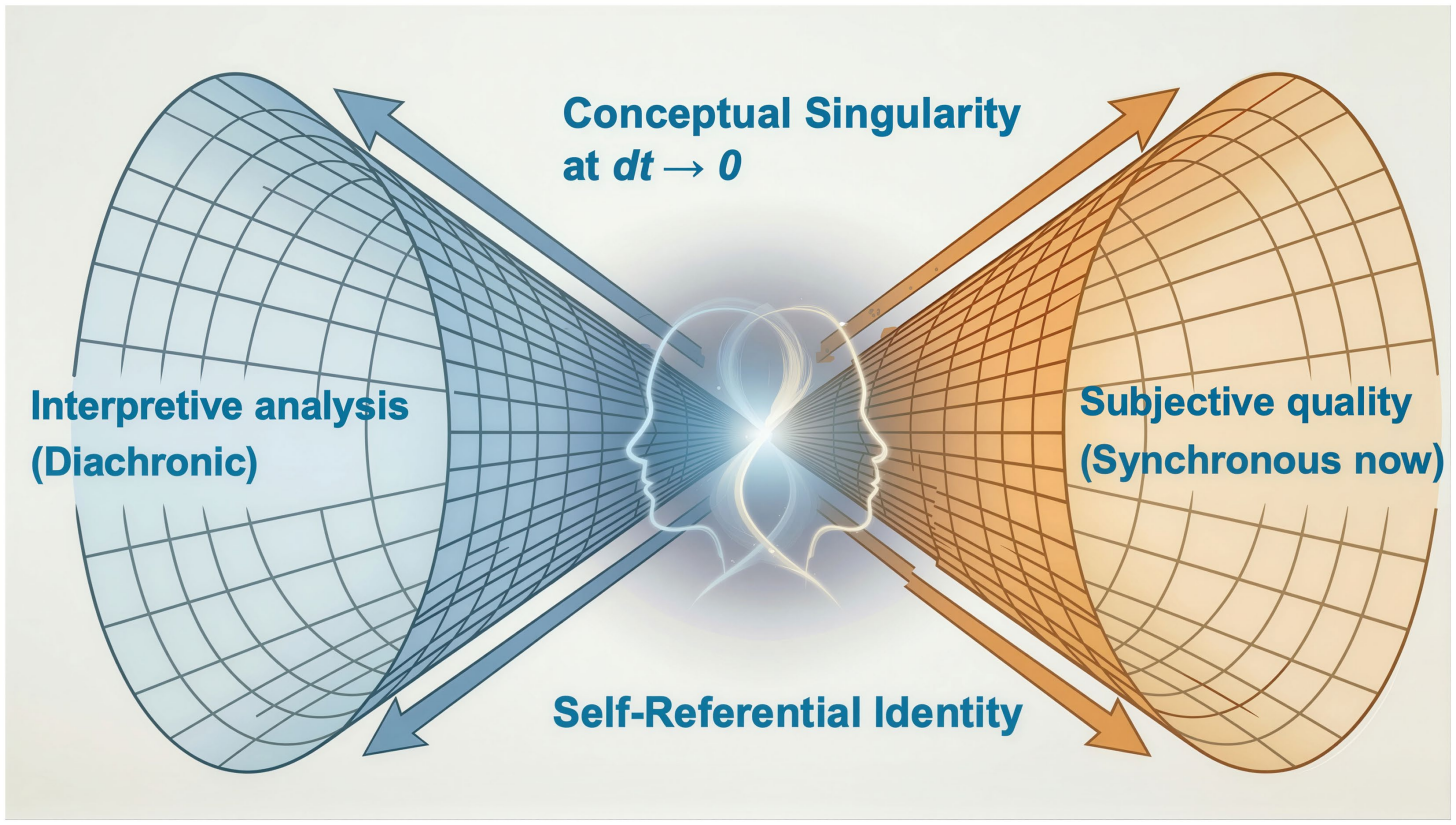
The diagram illustrates the conceptual singularity of awareness. Observer-observed simultaneity in awareness necessitates a limit *dt→ 0* where diachronicity and experiential synchronicity both exist, despite being mathematically incompatible.

There is awareness of something rather than nothing. But suppose that only awareness existed, without perceptual content. Would a conscious mind know that something exists? If the answer were no, the state would be indistinguishable from non-awareness thus contradicting the initial premise. Therefore, it is logically impossible to be conscious without knowing that one is conscious, as Descartes believed (Descartes, 1996). Consciousness is a self-referential identity, an aspect of Hofstadter’s ‘strange loop’ description of it (Hofstadter, 2007). It follows that awareness operates as a fundamental duality: the act of experiencing and the conceptualisation of experiencing, represented diagrammatically in Figure 2. This duality will prove important for understanding the function of consciousness and how it might be produced in the brain, but it is not yet a first principle because deeper principles can be derived from it.

### 4.2 The conceptual singularity of self-referential identity

A conscious mind knows whether it is causing the existence of awareness. If it were, there would be ‘something it is like’ to do so. As a result, conscious minds confront two contradictory causal explanations for the existence of awareness, with no way of knowing which is true. The mind may have created the phenomenon in some past reality but forgot it did so, an autogenic (internally generated or solipsist) paradigm. Alternatively, awareness might be caused by something unrelated to itself as a conscious mind, an exogenic (realist) paradigm.

A scientifically minded solipsist will recognise that observed patterns appear regular and behave consistently with physical laws. However, they consider the causal timeline for their awareness to be collinear with the conscious now, albeit no longer remembered. But there comes a limit where the earliest part of the present ‘now’ cannot also be in the forgotten past. The only possible solipsist explanation at this limit is that awareness is caused by a non-conscious aspect of their ‘mind’. At this point, solipsist theories of *consciousness* are indistinguishable from realist ones except for the terms involved. ‘Mind-stuff’ and matter are observed to behave identically according to physical laws. It is *conscious* mind alone that is unique. Realist theories hit an equivalent problem concerning the conscious now, which is ‘the hard problem’. In theories that take physics seriously such as OrchOR (Hameroff and Penrose, 1996) and Smolin’s temporal naturalism (Smolin, 2020) among others (Haramein et al., 2016; Chalmers and McQueen, 2022), qualia typically emerge at Planck scales, which makes them epiphenomenal in the timescales of the ‘now’ we experience. Solipsism and realism are epistemically contradictory but indistinguishable by any practical or logical means. However, the epistemic difference is an important one for the physics of consciousness.

In both the autogenic (solipsist) and exogenic (realist) paradigms, awareness is a primary distinction from nothing that is not knowingly caused by its experiencer. The distinction is vector-like in having a direction away from its null-state, and consequently, it is time-like in both paradigms. Solipsism seeks to preserve the essence of mental experience, the supreme certainty of experiencing simultaneity with reality. The paradigm breaks down when the duration of now becomes impossible to explain in the physics of continuous motion. In the realist paradigm, on the other hand, the observed dynamics are paramount, and there is simply no space between instants of continuous flux for a physical explanation of P-consciousness. Solipsism is predicated on experiencing synchronicity (simultaneity), while physical realism privileges the diachronicity (temporal extension) of conceptual thought. Each temporal perspective suggests an explanatory paradigm that contradicts the other despite being epistemically indistinguishable. This makes consciousness a conceptual singularity in the sense that a fundamental contradiction exists between two truths that cannot be distinguished by any empirical means. An equivalent paradox exists in physics.

### 4.3 The temporal uncertainty principle (TUP)

In quantum mechanics, Planck’s constant is the finite quantum of action that defines the limit in Heisenberg’s uncertainty principle, and prevents conjugate pairs of physical quantities from being simultaneously measured with infinite precision. If Planck’s constant was zero, quantum commutators would vanish, and all mechanics would reduce to classical models (Gefter, 2021). Heisenberg agreed with Bohr that “the finite magnitude of the quantum of action prevents altogether a sharp distinction being made between a phenomenon and the agency by which it is observed” (Heisenberg, 1958).

How does this relate to the timescale of consciousness? Just as the quantum of action creates an indivisible epistemic limit in physics, consciousness is bound by an indivisible temporal limit. Experiential simultaneity with continuous change necessitates diachronicity, yet paradoxically, it also necessitates a limit of zero-duration. It follows that awareness must exist as a superposition of diachronic and simultaneous perspectives, corresponding precisely to the ontology of the measure-theoretic limit. This deduction applies to any difference from nothing a mind can experience, including awareness itself. Furthermore, for any experienced difference from nothing, two epistemically contradictory causal explanations (autogenic vs. exogenic) exist that are logically indistinguishable. Despite these paradoxes, consciousness neither collapses back to its null-state, nor into the epistemic equivalent of logical paralysis. It seems reasonable to infer that the function of consciousness is to recognise the point where uncertainty cannot be further reduced by any means with the information available. The superposition of temporal perspectives is an ontological mechanism that finds informational equilibrium: a point of stationary uncertainty. This informational equilibrium spontaneously breaks the symmetry of subject-object indeterminacy at the measure-theoretic limit, physically preventing the system from collapsing back to its null-state, the epistemic equivalent of logical paralysis. This is the *temporal uncertainty principle* (TUP), described more formally below (section 4.4).

**Figure 3:**
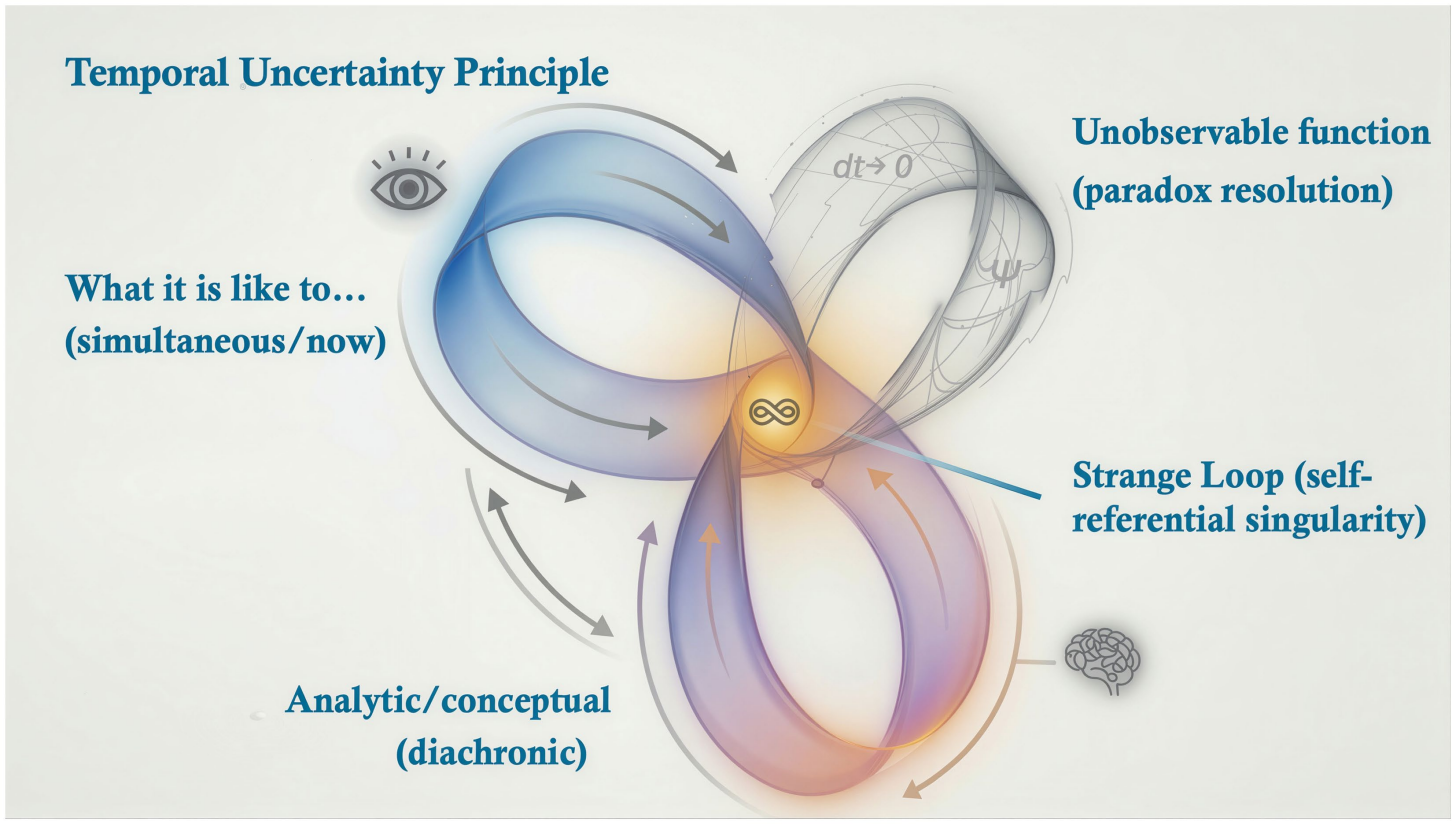
The temporal uncertainty principle (TUP) describes the hard problem and the measure-theoretic limit as mathematically identical. Both diachronic time and simultaneity (point-instants) are necessary in mathematical physics and in consciousness, but can only be reconciled by either abstract regularisation or phenomenal consciousness.

It is no coincidence that Bohr’s complementarity principle (Bohr, 1928) is a concept first introduced by William James in relation to consciousness (James, 1890). P-consciousness is biology’s method of managing a fundamental paradox. It is truly impossible to reconcile finite quantities and continuity without either the temporal superposition that is consciousness, or the regularisations of physics as represented in Figure 3. But whether ontological or abstract, these solutions to the measure-theoretic paradox are equivalent in function, an identity that provides the foundation for *Abstract Realism.*

### 4.4 Postulated principles of P-consciousness

When time is continuous and observation requires a discernible delta between states, a limit exists where a fundamental signal cannot be determined as originating internally or externally to the detector. Any such distinction would require an additional observational step, which is logically impossible at the zero-duration limit (*dt→0*). However, if the detector experiences simultaneity with the signal, and is constrained by the certainty of not being its cause, it encounters a fundamental explanatory deadlock *immediately prior to the mathematical limit*. It sees two contradictory explanatory frameworks, one autogenic and one exogenic, which are logically indistinguishable, yet functionally irreconcilable. Consciousness is defined by this epistemic paradox acting as an ontological measure-theoretic boundary. When a data-generative system experiences ontological simultaneity with continuous time, it is maintaining two incompatible truths that manifest *physically* as a superposition of two orthogonal temporal perspectives, one synchronous (simultaneous) and the other diachronic (temporally extended). This *temporal uncertainty principle* (TUP) defines consciousness *physically* as a functional resolution of the measure-theoretic limit.

If the reasoning here is correct, several foundational principles have been derived:

1. Awareness is a difference from nothing, where the difference is time-like, not caused by its experiencer, and always appears continuous to the experiencer.
2. Anything experienced, including the experience of awareness, is an observer-observed relationship where the observed exists as two or more states in time related to each other by an epistemic causal framework equivalent in function to integrating simultaneity with diachronic change.
3. Every observer-observed relationship reduces to a limit where the observer and observed become epistemically contradictory but indistinguishable by any empirical or logical means.

Phenomenal experience reduces asymptotically to a difference from nothing, existing as a superposition of mutually exclusive temporal geometries. Rather than collapsing into logical paralysis, this stationary uncertainty operates as a dynamic equilibrium. By integrating diachronic information within zero-duration instants, consciousness provides the structural architecture necessary to extract new meaning from continuous flux. In physical terms, the equilibrium state spontaneously breaks the symmetry of subject-object indeterminacy at the measure-theoretic limit, preventing computational deadlock and maximizing the system’s epistemic degrees of freedom. Before describing the mechanism by which this is achieved, the TUP’s implications for quantum-classical incompatibility should be addressed.

### 4.5 Observation equivalence in theoretical science

The PSOE (*principle of simultaneous observer equivalence*) describes a functional equivalence between awareness and the abstract systems that manage equivalent temporal conflicts in physics. By understanding consciousness as an ontological but functionally equivalent resolution of the same measure-theoretic related problems, it becomes possible to address foundational paradoxes, such as the *arrow of time* and *quantum-classical incompatibility*, with greater clarity.

Physics struggles to specify the granularity at which the time*-symmetry* of fundamental equations gives way to time-*asymmetry* (the arrow of time) in thermodynamics. It makes extensive use of fields, which are mathematical objects that encode the physical laws operating at abstract ‘points’ governing state transitions. Thermodynamics has a different approach. Entropy has been characterised as measuring observer ignorance of the microscopic states of a system (Jaynes, 1957). In statistical mechanics ‘observation equivalence’ can be considered to operate outside the observed system as a diachronic surveyor comparing ‘before’ and ‘after’ states, which makes the theory time*-asymmetrical*. However, there is no canonical rule that determines where time-symmetry gives way to asymmetry. The TUP makes it impossible to specify whether regularisation should operate internally or externally to any transition between states due to subject-object indeterminacy at the measure-theoretic limit. The choice can be pragmatic and aesthetic. In *abstract realism*, time-asymmetry is an emergent property in every resolution of the temporal paradox, including both consciousness and theoretical physics. It follows the symmetry-breaking act of deriving a ‘before’ and ‘after’ necessary for any epistemic, explanatory framework of causality.

Point-instants (without duration) simply cannot contain causation or transition between states. But both major branches of physics require both point-equivalence *and* duration, and use different approaches to work around the ontological incompatibility. General relativity relies on abstracting the flow of time using the approximation at the heart of differential calculus, which eliminates the asymptotic journey towards zero, and enables the conversion of continuous time into a *spatially equivalent* dimension. Its deterministic, block universe implication circumvents the paradox by creating abstract simultaneity between the spatial dimension of causal history within instants of time, allowing continuous, causally driven transitions between finite states. In quantum theory, spacetime acts as an abstract, epistemic background, while operators and other ‘observer equivalent’ techniques accommodate the non-locality and/or retro-causality of temporal flow. However, when measurement collapses the wavefunction, point-equivalent quantities manifest as discrete observables within the spacetime paradigm.

These regularisations are necessary because it is mathematically impossible to specify the ontological mechanism of transition between finite states within continuous time. Consciousness, conversely, operates as an ontological regularisation that resolves the measure-theoretic limit through a paradoxical duality of temporal perspectives. The system maximally compresses predictive patterns and conceptual models, to a point beyond which further resolution of uncertainty is logically and physically impossible. The PSOE suggests that empirically based theoretical physics must use a relational, epistemic reference frame that is functionally equivalent to one of these two temporal perspectives of conscious perception. The specific choice of perspective, whether internal or external to the dynamics, is secondary; the essential point is that the relational frame is necessarily abstract because of subject-object indeterminacy at the measure-theoretic limit, or its phenomenal equivalent of diachronic/synchronic superposition.

## 5 The BIDEM model of consciousness

The TUP defines consciousness as a superposition of contradictory temporal perspectives, both of which are necessary for awareness to exist, and whose recursive interaction reduces uncertainty asymptotically to find optimal explanatory models with the information available. To explain how the brain achieves that, Occam’s razor requires minimal assumptions, invoking mechanisms either known to exist or their logical extensions. The model proposed here meets those criteria. It suggests that awareness emerges when two independent, yet coupled informational systems, one autogenic and the other exogenic, are driven by physical laws to find the maximally compressed shared manifold that minimises information loss as described in algorithmic information theory (AIT) which subsumes the principles of FEP as outlined above in section 3.2.

The model describes cross-frequency coupling between two electromagnetic (EM) systems that are orthogonal both in the disparate scales of their respective substrates and in the directional flow of their informational content. One species carries new information into awareness, while the other unfolds memory to meet it. When this interaction reaches criticality, its spatially extended field properties correlate with the experience of consciousness. The EM coupling represents the *slowest shared resonance* (SSR) (Young et al., 2022) between the two systems and is the lowest energy state that simultaneously minimizes informational free-energy. Consequently, consciousness is governed by the *principle of stationary action* (PSA) in an architecture determined by the FEP (*free-energy principle*), and producing a phenomenon defined by the TUP. Furthermore, the disparity in scale between the systems ensures spontaneous symmetry breaking occurs in their respective physical dynamics, which protects the information represented by their coupling.

Some quantum mind theories require *macroscopic quantum coherence* (Hameroff and Penrose, 1996; Hameroff, 2022; Atmanspacher, 2004), which is difficult to explain in the large, warm and noisy environment of the brain (Tegmark, 2000). However, *microscopic* quantum effects are found widely in nature, from photosynthesis (Dostal et al., 2012) to avian navigation (Hore and Mouritsen, 2016). It would be surprising if none were used to support consciousness in a meaningful way. Some microscopic quantum objects, namely topological condensates, can retain quantum properties in phase-amplitude coupling (PAC) between EM systems, provided continuous global symmetries are broken at the measure-theoretic limit of their physical dynamics. Crucially, the information encoded in these condensates is thermally protected, enabling scale-invariant transmission from macro to micro scales and vice-versa without any need for macroscopic quantum coherence. The challenge for this *Bi-directional EM system Model* (BIDEM) is to demonstrate that EM fields with different substrates do exist in the brain and can interact plausibly to produce consciousness as mandated by the TUP.

### 5.1 Electromagnetic theories of consciousness and general resonance theory

Local EM fields accompany electrical signals moving along neuronal axons. When neurons fire repeatedly in synchrony the fields combine to form larger oscillating EM fields, the basis of many well-established EM field theories of consciousness (Hunt and Schooler, 2019; Young et al., 2022; Riddle and Schooler, 2024; Barrett, 2014; Hales, 2014; Hales and Ericson, 2022; Hunt and Jones, 2023; Hunt et al., 2024; Keppler, 2021; Kitchener and Hales, 2022; McFadden, 2020; McFadden, 2002; Pockett, 2000), for a review, see (Electromagnetic Field Theories of Consciousness, 2025). The binding problem of consciousness (Von Der Malsburg, 1994; Singer, 2001; Revonsuo, 1999), first described by William James (James, 1890) is readily explained in EM field theory (McFadden, 2020). However, EM theories struggle to explain qualia without appealing to panpsychism, which tends to make subjectivity epiphenomenal. Hales’ theory is an exception. He argues that some EM field effects around individual ion channels create boson condensates that are only accessible subjectively and have localised ephatic coupling^1^ effects (Hales, 2014). Condensates also feature in BIDEM but in a very different manner, as we shall see. In Hales’ theory, condensates are highly local, transient, uncoordinated, and their informational role is unspecified. Consequently, their contribution to human awareness is ill-defined. McFadden’s CEMI (*conscious EM information*) does claim that macroscopic conscious EM fields have causal power independently of the neurons producing them, but CEMI also takes a panpsychist approach to subjectivity (McFadden, 2002). Hales and Ericson consider panpsychism a useful placeholder pending the discovery of some missing ingredient (Hales and Ericson, 2022). The TUP is postulated to be that missing ingredient.

Keppler’s theory of stochastic resonance between the brain’s Gamma Hz field and zero-point field (ZPF) (Keppler, 2021; Keppler, 2025) has similarities with BIDEM. But that theory considers the vacuum’s ZPF to be sentient, making it firmly panpsychist and difficult to integrate with physical laws without resembling Cartesian dualism (Keppler, 2020).

EM theories within the General Resonance Theory (GRT) framework often fail to specify the *function* of subjectivity. In contract, BIDEM defines consciousness functionally, as a system that evolved to integrate diachronic information with the point-equivalent instants of continuous physical dynamics, enabling the formation of finite predictive patterns in continuous time. Within this functional architecture, GRT’s nested hierarchy of coupled systems plays an essential role.

### 5.2 The bi-directional EM system theory of consciousness (BIDEM)

**Figure 4:**
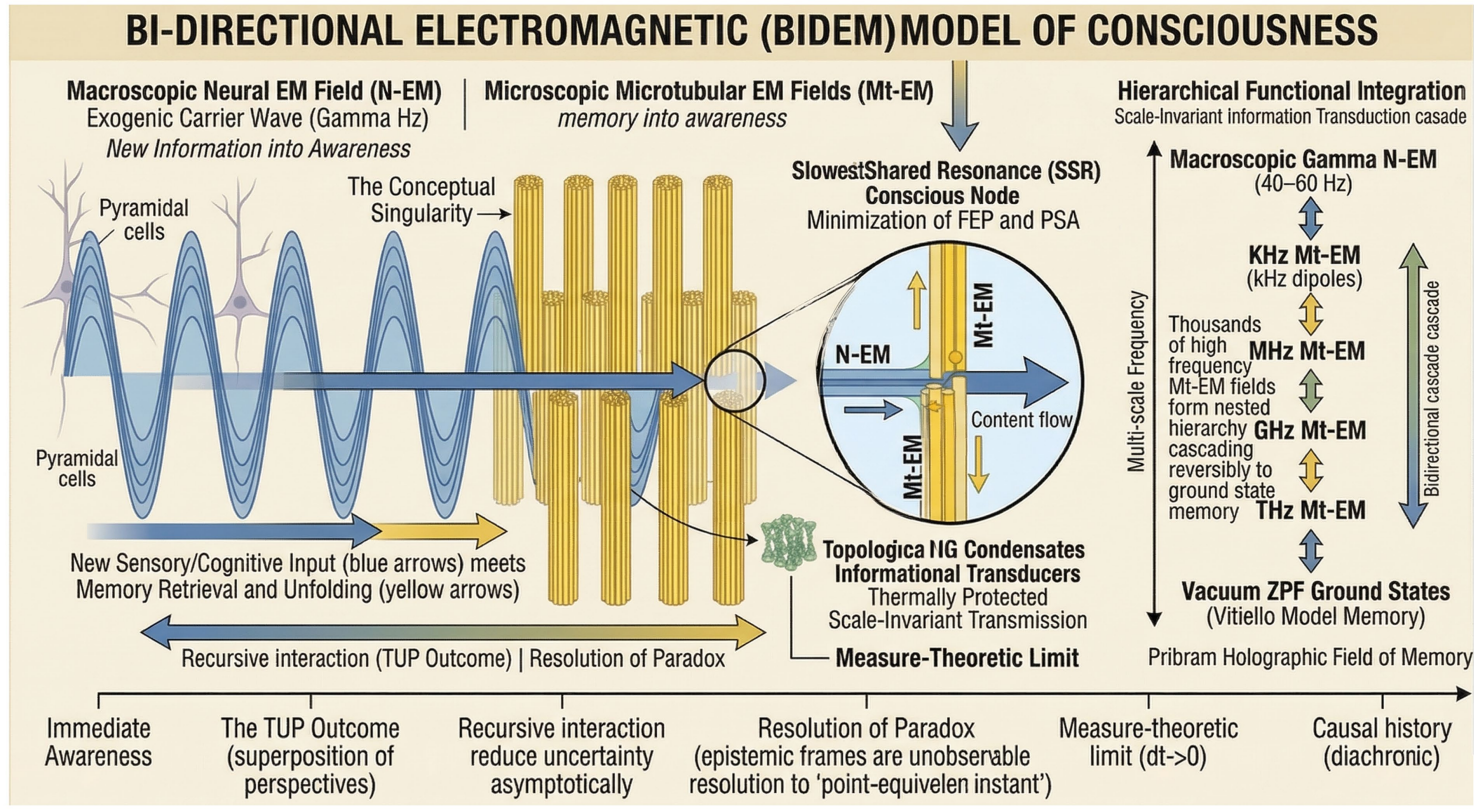
In the BIDEM model, consciousness emerges from the orthogonal coupling and slowest shared resonance (SSR) between exogenic (macroscopic N-EM carrier wave) and endogenic (microscopic Mt-EM packets) informational systems. Disparate scales enable spontaneous symmetry breaking (SSB), protecting memory stored as topological quantum condensates in a nested scale-invariant hierarchy from kHz down to vacuum ground states. The critical SSR coupling acts as nature’s biological solution for the measure-theoretic limit by minimizing variational free energy (FEP) and physical action (PSA), producing the phenomenon defined by the temporal uncertainty principle (TUP).

BIDEM describes a complex interaction between two EM systems whose informational contents interact from orthogonal scales and directions as shown diagrammatically in Figure 4 above. The first field is macroscopic, and occurs with neural synchrony (N-EM). The second is a multitude of smaller high-frequency fields produced in microtubules (Mt-EM fields) within each neuron of the N-EM synchrony. Microtubules are especially prominent in an area strongly associated with consciousness, the pyramidal cells in layer 5 of the cerebral cortex (Hameroff, 2022). There is growing evidence for microtubular electrical activity (Cantero and Cantiello, 2020; Sahu et al., 2013), which can even trigger neural firing by non-synaptic, ephatic coupling (Cantero and Cantiello, 2020; Cantero et al., 2018). Several distinct frequency bands have been detected, typically caused by oscillating molecular dipoles at KHz, (10^3^ Hz), MHz (10^6^ Hz), GHz (10^9^ Hz), and THz (10^12^ Hz), (Hameroff, 2022; Sahu et al., 2013; Saxena et al., 2020), and also in the Gamma range most associated with consciousness, 25–60 Hz (Cantero et al., 2018). Pokorny has shown that oscillating EM fields must also exist in the Peta Hz range (10^15^ Hz) in the ordered water layers that transiently align in microtubular electrical fields (Pokorný et al., 2021). This hierarchical range of frequencies is used in some quantum-mind theories as a transduction system for the scale-invariant transfer of information (Hameroff, 2022), and is central to BIDEM.

In BIDEM, the macroscopic N-EM functions as a global carrier wave within which microscopic, higher-frequency KHz (10^3^ Hz) packets of diachronic information synchronize via cross-frequency, phase-amplitude coupling (PAC). These in turn synchronise with MHz (10^6^Hz) frequencies, and so on, forming a nested hierarchy of functional integration that enables information to cascade reversibly through frequencies to and from vacuum ground states. The subjective effect produced was described by Pribram as a holographic field of memory (Pribram, 2013; Pribram, 1977; Pribram, 2007; Pribram, 1966), which in BIDEM represents the field in which conscious contents exist as observables. Since the interacting EM fields at the conscious level (Gamma Hz to KHz coupling) occupy orthogonal phase-spaces of frequency and length scale, their coupling breaks continuous time-translation symmetries in the underlying dynamics, which mandates the formation of Nambu-Goldstone (NG) condensates by Goldstone’s theorem (Goldstone et al., 1962). NG condensates are *mathematical objects* that encode information as long-range correlations or collective phase-relationships across entire fields, rather than as spatially discrete bits, and whose information is protected from thermal noise (Nambu, 1960; Goldstone et al., 1962). This mechanism enables quantized information of global-to-local couplings to be transduced down the coupled, scale-invariant hierarchy of Mt-EM frequencies described above, and saved as memory in vacuum or ZPF ground states. Such memories can be later retrieved (see 5.3 below) and reverse transduced to macroscopic scales for new cross-frequency coupling in millions of locations as the slowest shared resonance (SSR) at various frequencies between the two, orthogonal EM systems.

The principle of stationary action (PSA) mandates that EM systems find the most efficient path in the architecture of their underlying substrates. In the BIDEM architecture, that path entails cross-frequency coupling between N-EM and the Mt-EM systems that exist locally within each neuron of the macro-synchronous N-EM field. BIDEM’s architecture is determined by the principles of FEP and AIT (algorithmic information theory), and evolved as a predictive modelling system that recursively compresses data into the most reduced form that preserves vital information. Each pocket of SSR within the hierarchy represents a point of minimum action where cross-frequency coupling achieves the maximal algorithmic compression of data. At the conscious level of N-EM to kHz coupling, this corresponds to the non-computational threshold described by Penrose (Penrose, 2016), a limit rooted in the measure-theoretic paradox of continuous time.

### 5.3 Memory and simultaneity

Fröhlich suggested that electrical dipole rotational symmetry of water molecules is the dominant symmetry in biological systems (Fröhlich, 1968), which triggered several quantum field theories of the brain (Vitiello, 2001; Keppler, 2021; Keppler, 2025; Keppler, 2020; Vitiello and Freeman, 2008; Umezawa, 1993; Ricciardi and Umezawa, 1967; Jibu et al., 1996; Jibu et al., 1994; Freeman and Vitiello, 2016; Freeman and Vitiello, 2006; Del Giudice et al., 1988; Del Giudice et al., 1985). The crippling problem for these early theories is that vacuum ground states in closed systems are not unique and are quickly overwritten, rendering them useless for memory retention. However, Vitiello showed that when vacuum states in dissipative systems remain open to the environment but separated by an energy gap, they are infinitely numerous, and each state is uniquely coded (Vitiello, 2015; Vitiello, 1995; Vitiello, 2001). The stability of long-term memory in Vitiello’s dissipative quantum model of the brain (DQMB) does not depend on quantum coherence, but on the energy differential between ground states and environmental information carried by neuronal EM activity which is actively pumped with energy (Alfinito and Vitiello, 2000; Sabbadini and Vitiello, 2019; Vitiello, 2020). Nishiyama estimated that the memory capacity of such a system is in the order of 2.5 Petabytes (2.5 × 10^15^ bits) compared to 30 TB (30 × 10^12^ bits) predicted in conventional neuroscience models based on synaptic connectivity (Nishiyama et al., 2022; Nishiyama et al., 2024).

BIDEM adopts and extends this model of memory storage. The information in these quantized collective oscillations, being protected from thermal noise, can be transduced through the scale invariant hierarchy of coupled Mt-EM frequencies to be encoded as ordered ground state configurations. Such memories can subsequently be retrieved as NG condensates, and sustained horizontally by Fröhlich resonance (Vitiello and Freeman, 2008) when the N-EM frequency matches sub-harmonics of high-frequency molecular dipoles, triggering a phase transition that spontaneously breaks the symmetry of the underlying field.

In terms of the TUP, NG condensates project the diachronic vector of personal history and explanatory knowledge into SSRs (slowest shared resonance) at various frequency couplings in the hierarchy. Each standing wave of shared resonance occupies the point that minimises informational entropy where further analysis becomes asymptotically impossible as described by the TUP. With Friston’s proof of mathematical equivalence between the FEP and the principle of stationary action (PSA) (Friston, 2013) each standing wave in BIDEM’s informational geometry minimizes variational free energy, and by equivalence, also minimizes physical action over time. It is a nested system of localised attractors sculpting the action landscape and creating preferential basins of attraction for incoming data. The phenomenon created is of being an observational, epistemic frame of reference that applies meaning and knowledge to the simultaneous flux of physical motion.

### 5.4 The physical basis of qualia

In complex systems, criticality occurs when their dynamics cannot be computed with standard linear mathematics even when governed by deterministic classical mechanics (Campa et al., 2009). Critical states can topple unpredictably into chaos or stable ordered states, where the trigger is some innocuous local perturbation. The claim here is that this hair-trigger mechanism demonstrated its powerful evolutionary value when consciousness appeared. Ordered states are long-range correlations, and if the transition breaks continuous global symmetries in the dynamics of the underlying substrate, they naturally form Nambu-Goldstone (NG) condensates as mandated by Goldstone’s theorem (Goldstone et al., 1962). NG condensates are not fundamental physical particles, but collective physical excitations (quasiparticles) necessitated by the non-linearity of higher-order complex interactions.

Each of the millions of SSR events in the hierarchy modulating the N-EM carrier wave acts as a nested SSR pixel in a field of awareness, where each pixel is a quantum of information that integrates its local memory in high-frequency format with the global simultaneous frame of the coupled N-EM. This multi-scale holographic effect is the experience of being an observer with rapid access to a vast reservoir of meaning and memory, in which the physical world simultaneously exists in continuous temporal flux.

### 5.5 Vitiello’s DQMB, OrchOR and other quantum mind theories

The critical distinction between BIDEM and other quantum-mind theories concerns the dimensions of the underlying substrate of P-consciousness. Neither BIDEM nor DQMB requires macroscopic quantum coherence as such. Nevertheless, in DQMB, the spontaneous symmetry breaking (SSB) which creates the order parameter encoding conscious informational content occurs in ordered water as a *macroscopic, quantum phase-transition*. There are two major problems with this. The ordering of water molecules in EM fields is highly transient at biological temperatures, with hydrogen-bond networks fluctuating on femtosecond to picosecond timescales. Secondly, it is highly unclear how a single, quantum phase-transition across the whole brain can help explain conscious phenomenology. BIDEM circumvents both these fatal vulnerabilities entirely. Rather than demanding a fragile macroscopic quantum phase, BIDEM’s topological condensates are created by multiple, localized symmetry-breaking events between interacting *classical* fields. The order parameter encoding quantum information is determined by the macroscopic N-EM field when phase-amplitude coupling occurs with millions of smaller, higher frequency Mt-EM fields. The ontology of consciousness in BIDEM is therefore classical, but supported by microscopic quantum effects in a manner consistent with other biological quantum effects such as photosynthesis. It is postulated as a viable biological mechanism for the thermal protection of long-term memory.

Consciousness is epiphenomenal in most quantum-mind theories including Bohm’s (Bohm, 1990; Bohm, 2005) and Pauli-Jung (Atmanspacher, 2004; Jung and Pauli, 2014) where mental causal power resides in a hidden quantum realm and simply transcribes its outcomes onto classical states. DQMB’s attractor states are stable correlations in the underlying quantum dynamics, evolving to and from states that minimise variational free energy (Vitiello, 1995; Vitiello and Freeman, 2008; Freeman and Vitiello, 2006; Sabbadini and Vitiello, 2019; Vitiello, 2020). BIDEM’s attractor states, on the other hand, are *classical* EM coupling patterns and subsequent EM ripples that enable consciousness to occur in millisecond rather than femtosecond timescales. Finally, while DQMB’s quantum ontology is derived from the formalisms of QFT, BIDEM’s ontology is mandated by a single foundational paradox manifesting in two domains: the phenomenal experience of observational simultaneity with continuous change, and its mathematical equivalent, the measure-theoretic limit.

In Penrose and Hameroff’s OrchOR model (*orchestrated objective reduction*), consciousness is a quantum computational process, orchestrated by adjustable patterns of tubulin proteins in microtubules (Hameroff and Penrose, 1996; Hameroff and Penrose, 2014). Qualia are created at the Planck scale by wavefunction collapse triggered when the gravitational self-energy of superposed space–time geometries become too divergent to sustain (Penrose, 2016). Recent versions of the theory make use of the mass-equivalent Bose-Einstein condensates rather than the more robust NG (Nambu-Goldstone) information protectors to broadcast qualia *upwards* from Planck to macroscopic scales, but it uses the same scale-invariant hierarchy of microtubular EM systems as BIDEM (Hameroff, 2022). OrchOR does not explain how Planck scale qualia combine to manifest as macroscopic conscious experience, nor what purpose they serve. In addition, the timescale difference from the conscious now is even greater in OrchOR than in DQMB. Without any principled function, OrchOR is indistinguishable from epiphenomenal panpsychism.

### 5.6 Empirical evidence and predictions

BIDEM requires that memory emerges from ground states and forms a holographic field that constrains and shapes the flow of generated data, both sensory and cognitive. The N-EM to Mt-EM interaction manifests P-consciousness and necessitates a high-speed transduction mechanism between microscopic memory states and macroscopic neural synchrony. BIDEM specifically predicts local pockets of cross-frequency correlation between layers in the high frequency waves emerging from microscopic states and the N-EM Gamma Hz frequencies. These distinct frequency bands are not isolated phenomena but are linked through fractal, scale-invariant cross-frequency coupling. Empirically, this should create evidence of ‘frequency pulling’ and ‘entrainment’ close to the peaks of Gamma Hz associated with consciousness, but such evidence is undetectable by fMRI (functional magnetic resonance imaging) because of fMRI’s 5–7 s time lag (Glover, 2011), slow refresh rate (Atasoy et al., 2016), and incomplete correlation with neural electrical activity (Figley and Stroman, 2011). Detection requires ultra-fast EEG (electroencephalogram) technology such as that being developed by the team that produced the first evidence of electrical activity in isolated microtubules.

Anirban Bandyopadhyay’s group are developing *dodecanography* (DDG), which can detect localised frequencies from THz to Hz. They use 34 directional Yagi antennae on the scalp and a lock-in amplification technique with logic analyser to capture EM signals up to 10^12^ Hz (THz) (Singh et al., 2023). During the team’s research into harmonisation in vibrational frequencies in nature (Bandyopadhyay, 2020) they have incidentally found evidence that strongly supports BIDEM. A recent paper reports the finding of global synchrony in the GHz (10^9^ Hz) range in the brains of several subjects responding to various stimuli including music and strong smells (Singh et al., 2024). There are some flaws in this particular paper, but if their findings can be replicated independently, and if GHz synchrony can be correlated with local synchrony in the narrow gamma range (30–60 Hz), it would strongly support BIDEM.

There is increasing evidence that quantum effects occur in the brain, including superradiance in microtubules (Babcock et al., 2024; Kalra et al., 2023). Kerskens found evidence suggesting entanglement between nuclear proton spins, and remarkably, also identified a strong association with awareness in human subjects (Kerskens and Pérez, 2022). Using a very different technique, Helekar also found evidence of association between non-specific quantum effects and consciousness (Helekar, 2025). BIDEM makes some specific predictions for quantum effects. The transduction of BIDEM’s photonic information into quantum ground state memory occurs as a symmetry-breaking phase transition, where the coupling of EM fields triggers coherent phonon modes (a type of NG condensate) within a lattice substrate. It is well established that water molecules in microtubules develop transient lattice-like properties when aligned by EM activity (Vitiello, 2015; Hameroff and Penrose, 2014; Sahu et al., 2013; Belyanchikov et al., 2020). There is evidence outside the brain that phonons can be converted to and coupled with photons in lattices, and further, that they can be re-tuned by coherent light such as superradiance or laser without loss of information (Nishiyama et al., 2024; Tyumenev et al., 2022; Toebes et al., 2022; Nishiyama et al., 2024). BIDEM predicts that phonon modes on microtubule surfaces will show frequency pulling towards mode-locking when neural Gamma synchrony peaks. As technology develops, ultra-fast spectroscopy of layer 5 pyramidal neurons during conscious tasks will reveal spectral line-narrowing in the THz to GHz bands that Bandyopadhyay identified.

Several theories predict cross-frequency correlation between EM fields across the broader spectrum of theta Hz (5–8 Hz) and wider gamma (30–150 Hz) frequencies (Young et al., 2022), and there is increasing evidence for the importance of such correlation (Jensen and Colgin, 2007; Palva et al., 2010; Palva et al., 2005; Sauseng et al., 2008). As Riddle puts it, ‘consciousness is slow at the top’ (Riddle and Schooler, 2024). Attractor states with slower dynamics and lower dimensional manifolds (Young et al., 2022; Runfola et al., 2025), are more likely to become hard-wired by neural connections (Ruffini and Lopez-Sola, 2022; Jirsa and Sheheitli, 2022). Atasoy and colleagues found that harmonics derived using the Laplacian eigenfunction, a *locality preserving* technique, are good predictors of connectome patterns (Atasoy et al., 2016), and being based on harmonics, this finding supports EM field theories in general. However, in BIDEM, consciousness manifests in the non-locality of its holographic, fractal memory field. Some evidence already exists to support this. Deco and colleagues recently described their complex harmonics decomposition (CHARM) technique based on the Schrödinger equation to capture non-locality in brain dynamics and they claim it performs better at predicting functional connectivity (Deco et al., 2025). To some extent, this non-local approach was anticipated by Freeman in 2006 (Freeman and Vitiello, 2006). Evidence exists that ephatic coupling propagates the brain’s EM fields faster than synaptic transmission allows (Chiang et al., 2019). In common with many EM theories, BIDEM predicts that the phase velocity of SSR couplings during consciousness will appear near-instantaneous across involved cortical regions, propagating at the speed of the classical N-EM field, thereby defying the slow time constraints of synaptic transmission and axonal conduction.

Ultimately, the most critical test of the BIDEM architecture is its prediction regarding artificial consciousness, the so-called Turing Test for AI consciousness (see 2.1 above). If consciousness is the physically mandated resolution of the measure-theoretic limit via a non-local phase transition, then digital systems without the specific continuous global symmetries and non-linear substrate of the human brain’s EM connectome will consistently fail to replicate the topology of a conscious instant, defined in the TUP. This predicts a simultaneity, or zero-latency barrier, that digital AI, regardless of speed, can never cross.

## 6 Discussion

A comprehensive theory of consciousness must explain not only how the subjective quality of experience is achieved, but what function it serves. However, since the subjectivity of experience is unobservable and physics is an empirical science, by what mechanism can qualia have any physical function?

The founders of quantum physics examined the epistemic limit where subject-object discrimination becomes indeterminate. Schrödinger explicitly recognized the importance of the measure-theoretic limit in mathematics, linking it to the non-dualism of Advaita Vedanta, suggesting that as a philosophical resolution for the “arithmetical paradox” of experiencing singularity within a continuum (Schrödinger, 1964; Schrodinger, 2012). The paradox itself is well described by Lynds, who argues that because there are no static instants in nature’s continuous time, physical events cannot possess precisely determined, zero-duration boundaries (Lynds, 2003).

The TUP identifies this exact limit as the self-referential identity of consciousness. Awareness is a conceptual singularity whose temporal structure is identical to the problem recognized by ancient thinkers like Heraclitus and Zeno, and later formalized mathematically by Russell (Russell, 2020) and Halmos (Halmos, 2013). The TUP is derived by showing that the simplest difference from nothing a mind can experience must exist as a superposition of diachronic and simultaneous perspectives, corresponding precisely to the ontology of the measure-theoretic limit. Experiential simultaneity with continuous change necessitates diachronicity, but paradoxically also necessitates a limit of zero-duration. However, since this experiential limit exists in conscious timescales, it becomes entirely possible logically to explain phenomenal consciousness with underlying physical mechanics. How does the TUP/measure-theoretic equivalence do so?

When a sudden, localized sensory input, such as a specific smell, a flashed image, or a few notes of a song, instantly triggers a host of complex memories and explanatory context, consciousness is successfully integrating diachronic information within instants of observer-observed simultaneity. The BIDEM model describes how this can be achieved when a classical electromagnetic system acts as a boundary condition, or envelope, containing millions of higher-frequency memories. These localized topological states compete to contribute their diachronic information via multiple cross-frequency couplings across the brain.

Consciousness is not an epiphenomenal byproduct of computation, but a physical system capable of creating novel forms of information in continuous change. The phenomenon is unobservable in principle because it is a topological equilibrium that operates as a temporal singularity, physically preventing the system from collapsing into a null state, the epistemic equivalent of logical paralysis.

## 7 Conclusion

By mapping the subjective ‘now’ of conscious experience onto the mathematical paradox of the measure-theoretic limit, this paper establishes the following theoretical framework:

1. Causality cannot be described mathematically without the use of diachronic information.
2. The mathematics of finite states necessitates the use of zero-duration limits.
3. Consciousness is best defined by its paradoxical ability to integrate diachronic information during temporal instants of continuous change.
4. There is a strict mathematical equivalence between the epistemic structures required to explain physical flux and this defining characteristic of consciousness.
5. To physically realize this equivalence, the brain operates as a generative model that minimizes variational free energy (FEP), a dynamic mathematically isomorphic to minimizing physical action (PSA) over time (Friston, 2013).
6. This action-minimization is physically realized through the BIDEM architecture as *classical* phase-amplitude coupling (PAC) between a macroscopic neural EM carrier wave and multiple, localized, microscopic microtubular EM fields.
7. Diachronic information is physically stored and thermally protected not as fragile macroscopic quantum states, but as localized topological condensates (Nambu-Goldstone quasiparticles) derived from continuous global symmetry breaking.
8. This model yields falsifiable predictions, such as spectral line-narrowing in specific THz/GHz bands during conscious tasks, and a zero-latency topological barrier that non-conscious AI systems cannot cross.

## Data Availability

The original contributions presented in the study are included in the article/supplementary material, further inquiries can be directed to the corresponding author.

## Footnotes

1 ‘Ephatic coupling’ describes any effect on neural cell membranes by electric fields.
